# Serine protease RAYM_01812 (SspA) inhibits complement-mediated killing and monocyte chemotaxis and contributes to virulence of *Riemerella anatipestifer* in ducks

**DOI:** 10.1080/21505594.2024.2421219

**Published:** 2024-10-25

**Authors:** Rongkun Yang, Sen Li, Jie Guo, Yanhua Wang, Zeyuan Dong, Qing Wang, Hongying Bai, Congran Ning, Xiaotong Zhu, Jiao Bai, Sishun Hu, Yuncai Xiao, Zili Li, Zutao Zhou

**Affiliations:** a College of Veterinary Medicine, Huazhong Agricultural University, Wuhan, China; b Key Laboratory of Preventive Veterinary Medicine, in Hubei Province, Huazhong Agricultural University, Wuhan, China; c Hubei Hongshan Laboratory, Huazhong Agricultural University, Wuhan, China; d COFCO Nutrition and Health Research Institute, Beijing, China

**Keywords:** *Riemerella anatipestifer*, RAYM_01812, subtilisin-like serine protease, complement escape, virulence, poultry

## Abstract

*Riemerella anatipestifer* (RA) is a significant poultry pathogen causing acute septicemia and inflammation. The function of protease RAYM_01812, responsible for gelatin degradation, is unexplored in RA pathogenesis. To elucidate its role, we generated a deletion mutant ΔRAYM_01812 (ΔRAYM) and complementary CΔRAYM_01812 (CΔRAYM) strain and revealed the protease’s role in extracellular gelatinase activity. By expressing full-length 76 kDa RAYM_01812 protein without signal peptide as well as seven partial structural domains fragments, we evidence that the N-terminal propeptide acts as an enzymatic activity inhibitor and it gets cleaved at A^112^. Also, we show that the β-fold sheet domain is necessary for enhancing the enzymatic protease activity. Sequential auto-proteolysis forms a stable 40 kDa enzyme. Then, testing the strains in duck sera indicated that the absence or presence of RAYM_01812 results in reduced or enhanced bacterial survival, respectively. Furthermore, we found that the protease is able to cleave IgY antibodies as well as the complement factors C3a and C5a, that the protease reduces C3a- or C5a-mediated monocyte chemotaxis, and results in enhanced membrane attack complex (MAC) formation on the surface of ΔRAYM compared to CΔRAYM. This suggests that RAYM_01812 plays a crucial role in protecting against the serum complement-mediated bactericidal effect through inhibiting MAC formation and monocyte chemotaxis. Animal infection assays showed a 1090-fold reduced virulence of ΔRAYM compared to RA-YM, evidenced by decreased tissue loading and weaker histopathological changes. In conclusion, RAYM_01812 acts as a vital virulence factor, enabling host innate immune defence escape through complement killing evasion and monocyte chemotaxis inhibition.

## Introduction

The gram-negative, rod-shaped, non-spore-forming bacterium *Riemerella anatipestifer* (RA) – a member of the genus *Riemerella* of the family *Flavobacteriaceae* – causes acute septicemia and exudative inflammation in poultry, such as ducklings, geese, turkeys, and other domestic birds [1,2]. The high associated morbidity and mortality rates consequently cause serious economic losses in the global duck industry [3]. Currently, effective protective strategies – such as vaccine immunity – are missing, especially as cross-protection among the 21 RA serotypes described so far is negligible [4,5]. Thus, considering limits posed on antibiotic overuse in poultry production, innovative methods targeting RA infection are highly warranted.

The innate immune system complement – consisting of the classical pathway (CP), the lectin pathway (LP), and the alternative pathway (AP) – acts as an important defence against invading microbes [6,7]. Complement-mediated recognition of evolutionarily conserved pathogen-associated molecular patterns (PAMP) results in membrane attack complex (MAC) formation, release of C3a and C5a anaphylatoxins followed by phagocyte attraction, and subsequent lysis of the pathogens [8].

However, co-evolution with the host enabled pathogens to escape the bactericidal action of the complement system through capturing host regulatory factors, producing complement inhibitory factors, or cleaving complement components through secreted proteases – hydrolytic enzymes catalyzing peptide bond cleavage [9–12]. For example, secreted elastase (PaE) and alkaline protease (PaAP) – which mediate degradation of immunoglobulins (Igs), C1q, and C3a – enable *Pseudomonas* species to block the classical pathway of complement activation and thus to escape complement immunity. Moreover, the subtilisin-like serine protease C5a peptidase expressed by group A and group B *Streptococcus* species specifically cleaves human complement C5a [13–15], resulting in loss of anaphylatoxin-mediated chemotaxis [16–18]. Also, *Serratia marcescens* blocks C5a-mediated inflammatory and chemotactic responses by secreting a 56 kDa C5a-specific protease [19,20].

In general, secreted proteins play important roles in microbial pathogenesis. In fact, secreted members of the type IX secretion system (T9SS) enhance RA survival through inhibition of the classical and alternative complement pathways [21]. Also, we previously demonstrated that the RAYM_01812 protein is secreted through T9SS and affects RA-mediated pathogenesis through its subtilisin-like serine protease domain [21]. Moreover, a homologous protein of RAYM_01812 in *R. anatipestifer* strain Yb2 named SspA (secreted subtilisin-like serine protease) was previously shown to mediate virulence and defence against natural host immunity of *R. anatipestifer* by proteolysis of gelatin, fibrinogen, and LL-37 [22]. However, precisely how RAYM_01812 mediates complement escape of RA remains to be determined.

In the present study, we generated a RAYM_01812 gene deletion (ΔRAYM) mutant and complemented CΔRAYM strain to investigate the relationship between RAYM_01812 and the biological characteristics and virulence of RA. Furthermore, we expressed and purified the full-length protein as well as fragments thereof to determine self-proteolytic cleavage sites and to clarify how RAYM_01812 mediates complement system escape.

## Materials and methods

### Ethics statement

Animal experiments were accordant with the ARRIVE guidelines and recommendations of the Research Ethics Committee of Huazhong Agricultural University, Hubei, China (Approval No. HZAUSW-2018-011).

### Source of animals and housing conditions

A total of 12 30-day-old and 240 12-day-old Cherry Valley ducks were purchased from Wuhan Yongsheng Duck Farm (Hubei, China) and housed at the laboratory animal center of Huazhong Agricultural University. During the study period, 5 to 6 ducks were kept per cage in a 28 to 30°C environment with 12 hours of light daily, and the ducks were given antibiotic-free feed and water without restriction.

### Bacterial strains, plasmids, and culture conditions

Bacterial strains and plasmids used in this study are listed in Supplementary Table S1.

*R. anatipestifer* serotype 1 strain RA-YM was the wild-type strain, isolated from the brain tissue of a diseased duck and preserved in laboratory of veterinary microbiology and immunology of Huazhong Agricultural University [23]. RA strains were grown on tryptic soy agar (TSA) or broth (Difco, Detroit, MI, USA) at 37°C in 5% CO_2_. *Escherichia coli* was grown at 37°C on Luria-broth agar or in broth. When required, bacterial culture media were supplemented with ampicillin (Amp, 100 μg/mL), chloramphenicol (50 μg/mL), spectinomycin (Spc, 100 μg/mL), diaminopimelicacid (50 μg/mL), or kanamycin (Kna, 50 μg/mL). Chloramphenicol and Spc were used for the screening of the deletion strain ΔRAYM, Amp and Spc were used for the screening of the revertant strain CΔRAYM, diaminopimelic acid was used for the culture of χ7213, and Kna was used for the pET-28a expression vector.

### Construction of RAYM_01812 gene deletion mutant and complementary strains

Deletion of ΔRAYM occurred by allelic exchange using the recombinant suicide plasmid pRE112. Briefly, a 431-bp left flanking region of the RAYM_01812 gene (Left arm) was amplified from RA-YM genomic DNA using primers 01812-F1 (introducing a *Kpn*I site) and 01812-F2. A 1086-bp Spc^R^ cassette was amplified from plasmid pIC333 using primers Spc-F1 and Spc-F2. An 880-bp right flanking region of RAYM_01812 gene (Right arm) was amplified from RA-YM genomic DNA using primers 01812-R1 and 01812-R2 (introducing a *Sac*I site). The left flanking region, Spc^R^-cassette, and right flanking region were fused by overlap extension PCR using 01812-F1 and 01812-R2. The fused DNA fragment was inserted into the pMD18-T vector to generate pMD18T-LSR, which was digested along with the pRE112 plasmid with *Kpn*I and *Sac*I to obtain the recombinant suicide plasmid pRE112-LSR. Upon transformation into *E. coli* χ7213, the transformants served as donor strain for conjugal transfer. The deleted mutant strain was selected on Tryptic Soy Agar (TSA) containing 100 μg/mL Spc. The RAYM_01812 gene deletion mutant strain (ΔRAYM) was confirmed by PCR.

The shuttle plasmid pRES-JX-bla was constructed as previously described by replacing the replication region of plasmid pRE112 with the putative replication region of the RA plasmid RA-JX [21]. The promoter and coding sequences of RAYM_01812 were amplified using primers 01812-C1 (introducing an *Sph*I site) and 01812-C2 (introducing an *Xba*I site). The product was digested with *Sph*I and *Xba*I and inserted into pRES-JX-blah to construct the recombinant transport plasmid pRES-C01812. Adopting the combination transfer method, the complemented strain was screened on TSA with Spc and Amp. The RAYM_01812 gene complemented strain (CΔRAYM) was identified by PCR.

### Biochemical characterization of RAYM_01812 gene mutant strains

RA-YM, ΔRAYM deleted strain, and CΔRAYM complemented strain were grown for 48 h at 37°C in 5% CO_2_ on TSA plates and inoculated in TSB. At mid-exponential phase, the culture was diluted with fresh medium to an optical density at 600 nm (OD_600_) of 1.0. Biochemical testing of the three strains was done in bacterial biochemical tubes (Hope Bio, Qingdao, China) with glucose, arabinose, sucrose, citrate, hydrogen sulfide, nitrate, carbamide, and gelatin.

### Enzyme activity assay

The amino acid content was determined by ninhydrin colorimetry as previously described [24]. RA-YM, ΔRAYM, and CΔRAYM were inoculated in TSB medium at 1%, incubated at 37°C for 20 h while shaking at 200 rpm, and centrifuged at 12,000 rpm for 20 min at 4°C. Then, the supernatant was collected, filtered with a 0.22 μm filter to remove the bacteria, and stored at 4°C. Next, 0.5 ml of type I collagen (0.1 M/L Tris-HCl (pH 7.5), 10 mm CaCl_2_) was mixed with 0.1 ml of bacterial culture supernatant, incubated for 20 min at 37°C, after which the reaction was terminated with 10% trichloroacetic acid. The water-soluble amino acids and short peptides released from the reaction were determined by ninhydrin colorimetry while a standard curve was developed by glycine coloration. One enzyme activity unit (U) was defined as the amount of enzyme equivalent to 1 μg of glycine produced by hydrolysis of collagen per minute at 37°C and pH 7.5.

### Cloning, expression, and purification of segmented fragment of RAYM_01812, duck C3a, and C5a

The coding sequences of the different RAYM_01812 fragments (NCLβT (without the signal peptidase), NC, NCL, NCLβ, NCT, NCLT, CD, and β) as well as the duck C3a and C5a genes were amplified by PCR (primer sequences are shown in Supplementary Table S1). The amplified fragments were digested with *BamH*I and *Xho*I and ligated in vector pET-28a and transformed into *E. coil* strain BL21 (DE3). Recombinant proteins were purified as previously described [24].

### Construction of catalytic residue mutants Ser410Ala and RAYM_01812 (S410A) protein expression purification

The primer pair S410A-F/S410A-R – designed on both sides of the mutation site and overlapping with each other by 29 bp – as well as the full-length primer pair of RAYM_01812 were used on 0.1 μg pET-28a-RAYM_01812 recombinant plasmid as template. The primer pair NCLβT-F and S410A-R guided the synthesis of the fragment upstream of the mutation site (fragment A), while the primer pair S410A-F and NCLβT-R guided the synthesis of the downstream fragment B. Purification of fragment A and B was followed by PCR on the overlapping templates of fragments A and B with the primers NCLβT-F/NCLβT-R. The recovered product was ligated in pET-28a (+) vector to create the recombinant mutant plasmid pET-28a-RAYM_01812(S410A). The sequence was verified by *BamH*I/*Xho*I digestion and sequencing (Sangong Biotechnology, Shanghai). The plasmid was transformed into BL21(DE3) E. *coli* for RAYM_01812(S410A) protein expression and purification [22]. The resulting gelatinase activity was determined as described in the text.

### Gel electrophoresis, zymography, and N-terminal sequencing

The purified NC, NCL, NCT, NCLT, CD, β, NCLβ, and RAYM_01812 (NCLβT) proteins were incubated at 37°C for 0, 0.5, 1, 4, 8, 12, 24, 48, 72, 96, 120, 144, and 168 h. Protein purity was evaluated by 10% SDS-PAGE. The gel was stained with 0.1% Coomassie brilliant blue *R*-250 in 10% acetic acid and destained in 30% methanol, 10% acetic acid.

Gelatin zymography of the purified RAYM_01812 protein was performed as previously described [24]. The protein was incubated at 37°C for 0, 0.5, 1, 4, 8, 12, 24, 48, 72, 96, 120, 144, and 168 h and then mixed with non-reducing SDS sample buffer (1:1). The mixture was resolved by 10% SDS-PAGE on gels containing gelatin at a final concentration of 1 mg/mL. Gelatinolytic activity was visible as unstained zones against a blue background.

The purified RAYM_01812 protein was incubated at 37°C for 168 h. Proteins were subjected to SDS-PAGE and transferred to a polyvinylidene difluoride membrane. Cleavage products were visualized with 0.1% Coomassie blue in 40% methanol, excised, and analyzed by N-terminal sequencing (Alphalyse).

### Serum survival assay

Blood was collected from 12 healthy 30-day-old Cherry Valley ducks without anti-RA antibody and immediately placed on ice. After 2 h, the clotted blood was centrifuged at 2000 g for 10 min at 4°C, the serum was collected, and stored at −80°C.

For the serum survival assay, the deleted strain ΔRAYM, the complemented strain CΔRAYM, and the wild-type strain RA-YM were cultured in TSB to an OD_600_ of 0.8. The cells were washed, resuspended to 10^6^ CFU/mL in Hank’s Balanced Salt Solution (HBSS) (Gibco, Grand Island, NY, USA) with 0.15 mm Ca^2+^ and 1 mm Mg^2+^ (HBSS++), and incubated at 37°C for 30 min with or without duck serum. Then, 10-fold serial dilutions were spread onto TSA plates followed by incubation for 48 h and colony counting. To evaluate the viability of all strains in the serum survival assay, all bacteria resuspended in HBSS++ to prevent replication [21] or in the presence or absence of 10 mm Mg^2+^ EGTA to block the classical and lectin complement pathways and selectively activate the alternative complement pathway. Heat-inactivated serum used in this assay was generated by incubating normal duck serum at 56°C for 30 min. The survival rate was calculated as (number of cells that survived serum treatment/number of cells that survived control treatment) × 100%.

### Serum survival assay of RAYM_01812 protein mediated *E. coli* DH5α

*E. coli* DH5α was cultured in LB to an OD_600_ of 0.8 and 10-fold serially diluted to 10^6^ CFU/mL in Hank’s Balanced Salt Solution (HBSS) (Gibco, Grand Island, NY, USA). Healthy duck serum was treated with or without heating at 56°C for 30 min. Serum was preincubated with different concentrations of RAYM_01812 protein for 30 min at 37°C in HBSS. Subsequently, DH5α was added and incubated for 30 min at 37°C. Next, cells were 10-fold serially diluted, spread onto LB agar plates, and incubated for 24 h at 37°C. The survival rate was calculated as (number of cells that survived serum treatment/number of cells that survived control treatment) ×100%.

### Duck immunoglobulin IgY cleavage test by RAYM_01812

A 20 μL system consisting of cleavage buffer (100 mm NaCl, 100 mm Tris base, 5 mm CaCl_2_, pH 8.5), 10 μM substrate protein IgY, and the indicated concentration of RAYM_01812 enzyme protein was incubated at 37°C for 2 h. Following SDS-PAGE, the bound IgY was detected with rabbit anti-duck IgY (Beijing Baiaolaibo Technology Co., Ltd, Qingdao, China) (1:6000) and peroxidase-labelled goat anti-rabbit IgG (1:4000) (Beijing Baiaolaibo Technology Co., Ltd, Qingdao, China) [25].

### MAC deposition assay

RA-YM and ΔRAYM were prepared using the conditions outlined above. Normal duck serum (NDS, final concentration 5%) was added to 10^8^ CFU of cell suspension and samples were incubated at 37°C. Aliquots were taken at 10, 20, 30, 40, 50, and 60 min and transferred to ice to terminate the complementation reaction. Then, samples were denatured in loading buffer at 95°C for 10 min, loaded on SDS-PAGE, transferred to PVDF membranes at 350 mA for 5 h, and visualized with rabbit anti-duck C9 (1:1000 dilution) in combination with HRP goat anti-rabbit IgG (1:10000 dilution) antibodies as described previously [26]. Enhanced chemiluminescence was used to detect signals and assess differences in C9 monomer and oligomer formation on the bacterial surface.

### Fluorescence resonance energy transfer test

Short duck C3a and C5a peptides were labelled with fluorescent groups at amino acid positions 62–77 and positions 62–75, respectively, as the substrates for the cleavage assay. Fluorescent substrate DUCK-C3a or DUCK-C5a (10 μM) was mixed with 1 or 2 μM RAYM_01812 protein or 5 mm PMSF in 200 μL cutting buffer (100 mm NaCl, 100 mm Tris base, 5 mm CaCl_2_, pH 8.5) and the reaction was carried out at 37°C for 2 h. Finally, the change of fluorescence value was determined with a multifunctional enzyme labelling instrument with excitation at 340 nm (35 nm) and emission at 485 nm (20 nm). The fluorescent substrates used are shown in Supplementary Table S2 and were synthesized by Nanjingjinsirui Science & Technology Biology Corp. Three replicates per group were set up in the experimental and control groups.

### Monocyte chemotaxis test

Fresh peripheral blood of healthy ducks was collected according to the kit instructions and single-nucleated cells were isolated. Duck C3a and C5a (10–20 nM) and RAYM_01812 protein (0–100 nM) were incubated at different concentration gradients for 2 h at 37°C. Then, the incubated proteins were added to the lower chamber of the chemotaxis chamber. After the isolated single nucleated cells were adjusted to 1 × 10^6^ cell/mL, 100 μL of cell suspension was added to each well in the upper chamber followed by incubation at 37°C for 90 min at 5% CO_2_. At the end of culture, the cells in the upper chamber were discarded, those on the upper surface of the 8 μm chemoadhesive membrane were removed with a cotton swab, and those attached to the lower surface of the chemoadhesive membrane were stained with 1% crystal violet. The number of cells was counted using a microscope at 200× [25].

### Evaluation of virulence of mutant strain ΔRAYM and complemented strain CΔRAYM in vivo

Bacterial LD_50_ of the mutant ΔRAYM01812-, the complemented CΔRAYM-, and wild-type RA-YM strain were measured as previously described. In brief, 12-day-old Cherry Valley ducklings were randomly divided into 3 groups (*n* = 60), injected intramuscularly (to ensure 100% infection of the experimental animal) with bacteria at 1.0 × 10^5^, 1.0 × 10^6^, 1.0 × 10^7^, 1.0 × 10^8^, or 1.0 × 10^9^ CFU (12 ducks per concentration). Mortality was recorded daily for 7 days, and LD_50_ was calculated using an improved Karber’s method [21].

To evaluate bacterial invasion into organs, the bacterial load in the blood, spleen, liver, heart, and brain was examined in pathological lesions. For histopathological analyses, 12-day-old Cherry Valley ducklings were randomly divided into three groups (*n* = 20), injected intramuscularly with 1.0 × 10^7^ CFU ΔRAYM-, RA-YM bacteria, or no treatment (control group). Measures were taken to prevent cross-contamination during the experiments. The organs and blood from ducks displaying morbid symptoms were collected at 24 or 48 h post infection, diluted appropriately, and plated on TSA plates. Colonies were counted with the plate pouring method. Spleen, liver, heart, and brain tissue sections were immersed in 10% formalin solution for subsequent histopathology.

### Statistical analysis

Statistical analyses were done in SPSS v.18.0 (SPSS Inc., Chicago, IL, USA) and Prism version 6.0 (GraphPad, La Jolla, CA, USA). Data for protease activity, chemotaxis test, FRET, and bacterial load in blood and serum in the survival test were analyzed using a two-tailed independent Student’s t-test. Data for the serum survival assay were analyzed by analysis of variance. Data are presented as mean ± standard error of the mean, and statistical significance was determined with the Student’s t-test. The significance level for all analyses was set as *P <0.05, **P < 0.01 and ***P <0.001.

## Results

### The RAYM_01812 protein is the only extracellular gelatinase of RA

**Figure 1.**
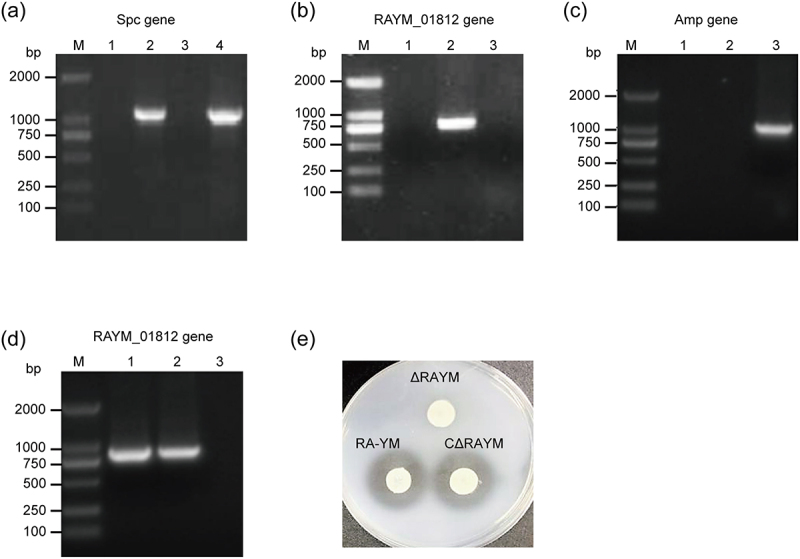
Identification and characterization of mutant strain ΔRAYM and complemented strain CΔRAYM. (a) Spc gene PCR amplification. Lane M: DL2000 DNA marker; lane 1: negative control (PBS); lane 2: positive control (plC333); lane 3: RA-YM; lane 4: ΔRAYM. (b) RAYM_01812 gene PCR amplification. Lane 1: negative control; lane 2: RA-YM; lane 3: ΔRAYM. (c) Amp gene PCR amplification. Lane 1: RA-YM; lane 2: ΔRAYM; lane 3: CΔRAYM. (d) RAYM_01812 gene PCR amplification of complemented strain CΔRAYM, mutant strain ΔRAYM, and wild-type strain RA-YM. Lane 1: RA-YM; lane 2: CΔRAYM; lane 3: ΔRAYM. (e) Loss of gelatinase activity from ΔRAYM mutant whereas wild-type RA-YM and CΔRAYM complemented mutant show gelatinase activity.

The mutant strain ΔRAYM was isolated by screening for Spc^R^ cassette-enabled growth on TSA plates. Subsequent PCR amplification confirmed the presence of one Spc fragment insert and the absence of the deleted part in ΔRAYM (Figure 1a). Similarly, the identity of complemented strain was also confirmed by PCR (Figure 1b-d).

All three strains (RA-YM, CΔRAYM, and ΔRAYM) were negative for glucose, arabinose, sucrose, citrate, hydrogen sulfide, nitrate, and carbamide. Whereas RA-YM and CΔRAYM were able to liquefy gelatin, ΔRAYM was not (Supplementary Table S3). The biological characteristics test suggested that there was no difference between the wild-type RA-YM-, the mutant ΔRAYM-, and complemented CΔRAYM strain, except in gelatin liquefaction features (Figure 1e). To further determine how much the enzyme activity of ΔRAYM was reduced, the gelatinase activity was measured in culture supernatants in which RA-YM, ΔRAYM, or CΔRAYM strains were cultured. Interestingly, whereas the gelatinase activities of RA-YM and CΔRAYM were 13.6 U/mL and 12.1 U/mL, respectively, that of ΔRAYM was 0 U/mL (Table 1). These data indicate that RAYM_01812 is secreted by RA and functions as an extracellular gelatinase.

**Table 1.** Culture filtrate gelatinase activity.

strain	RA-YM	ΔRAYM	CΔRAYM
gelatin liquefaction activity (U/mL)	+	−	+
	13.6	0	12.1

### RAYM_01812 is an auto-proteolytic serine protease

**Figure 2.**
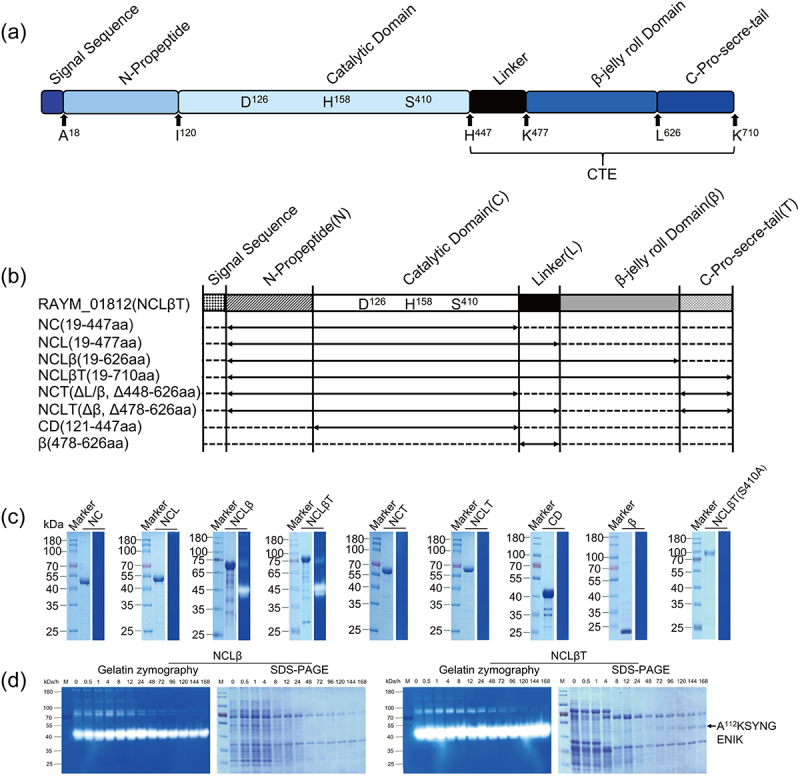
Automatic processing of different proteins and analysis of enzyme activity. Predicted RAYM_01812 protein domain structure of the S8 family of serine proteases in RA (a). Signal peptides were predicted using the signal IP 3.0 server. Predicted multidomain structure of RAYM_01812 and schematic diagram of segmentation (b). SDS-PAGE detection of purified proteins and ability of purified protein to degrade gelatin (c), proteolytic autoprocessing (d). Full-length RAYM_01812 and NCLβ were incubated at 37°C. At defined time points, aliquots were withdrawn for SDS-PAGE analysis followed by zymography with gelatin. The N-terminal sequence of the active band at 40kD was determined (d).

RAYM_01812 –annotated as a C5a peptidase in the RA-YM genome – contains an 18 bp signal peptide (SP), a unique N-terminal propeptide (NTP), a catalytically active structural domain (CD) with great similarity to the S8 serine protease family, and a C-terminal extended structure (CTE) containing the pre-linker region, the β-folding sheet, and the CTD region (Figure 2a). While the CTD structure mediates protein export by T9SS, the functions of the linker and β folding sheet are unknown. To determine the minimum enzyme activity unit as well as the functions of the RAYM_01812 domains, we expressed and purified the NC, NCL, NCLβ, NCLβT (RAYM_01812), NCT, NCLT, CD and β-fragments and performed a gelatin zymography assay (Figure 2b,c). Interestingly, whereas NCLβ and NCLβT exerted gelatinase activity, the NC, NCL, NCT, NCLT, CD, and β-fragments did not (Figure 2c). Meanwhile, the NCLβ and NCLβT fragments were incubated at 37°C for 168 h while samples were collected at different time points for SDS-PAGE followed by gelatin enzyme profiling. Following 30 min incubation, the 75.5 kDa band was weakened after which it sharply decreased to the observed level at 4–8 h. Meanwhile, a 40 kDa protein band gradually appeared while the 35 kDa protein band remained visible. Similarly, the gelatin and caseinase spectra showed the enzyme activity-mediated formation of a 40 kDa fragment. Obviously, the spectra and the SDS-PAGE results showed identical sizes of the active bands at overlapping time points (Figure 2d). To further clarify the self-proteolytic site of RAYM_01812, the 40 kDa band was sequenced by N-terminal sequencing and identified as a RAYM_01812-derived polypeptide truncated at the N-terminal A112 of the full-length protein (Figure 2d).

### RA complement escape correlates with RAYM_01812 protein

**Figure 3.**
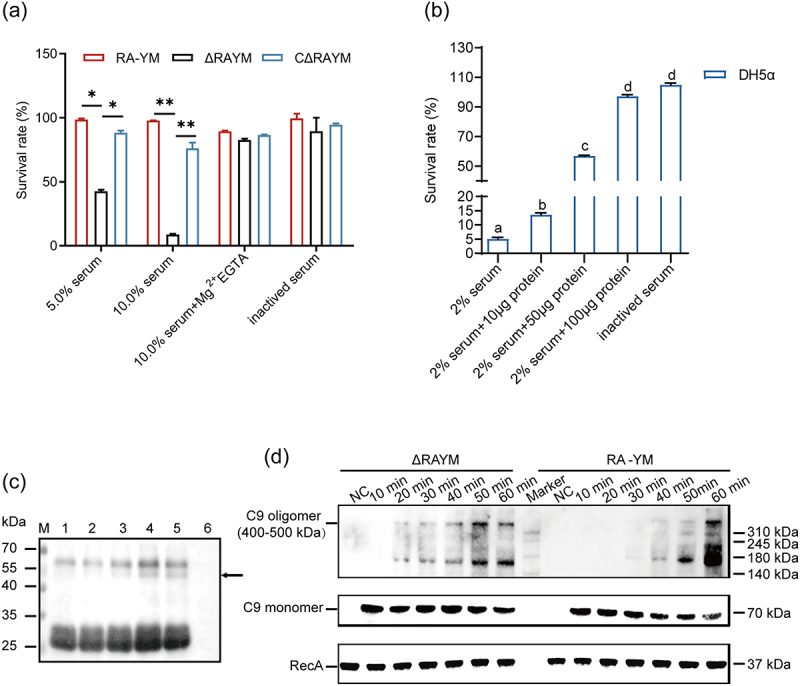
RA complement escape correlates with RAYM_01812 production. (a) Serum survival of wild-type strain RA-YM, mutant strain ΔRAYM, and complemented strain CΔRAYM. Data were analyzed with Student’s *t*-test (* *p* < 0.05, **p < 0.01). (b) The survival rate of *E. coli* DH5α in normal duck serum and RAYM_01812. Same letter means not significant difference (*p* > 0.05, while different lowercase letters refer to significant difference (*p* < 0.05). (c) Cleavage of immunoglobulin IgY by RAYM_01812. Lanes: 1, 10 μM IgY (10 μM) control; 2, PMSF (5 mm) inhibited the cleavage of IgY (10 μM) by RAYM_01812 (10 μM); 3, cleavage of IgY (10 μM) by RAYM_01812 (1 μM); 4, cleavage of IgY (10 μM) by RAYM_01812 (2 μM); 5. RAYM_01812 (10 μM) cleavage on IgY (10 mμ); 6, RAYM_01812 negative control. Note: observed IgY degradation products are indicated by arrow (→). (d) Western blotting assay of C9 deposition on RA-YM and ΔRAYM_ 01812 when incubated in 5% NDS for the indicated time periods. Three independent experiments were conducted and a representative experiment is shown here. The nature of data points belongs to biological repetition.

The ability of the mutant ΔRAYM- and wild-type RA-YM strain to resist complement-mediated killing was compared with a serum survival assay. To exclude the potential effect of variable growth rates among the two strains, the cells were resuspended in HBSS++ –preventing replication without affecting viability – and mixed with different duck serum concentrations. Interestingly, whereas the survival rate of RA-YM in 5% and 10% normal duck serum was 98.6% and 97.8%, that of ΔRAYM was 42.6% and 8.8%, respectively. Of note, the survival rate of the complemented strain CΔRAYM was comparable to that of the wild-type. Addition of Mg^2+^/EGTA to 10% normal duck serum preserved the survival rate of ΔRAYM to 82.6%. Consistent with complement-mediated killing, heat inactivation abrogated the bactericidal effect of serum in ΔRAYM. Together, these results indicated that RAYM_01812 is required for complement resistance in wild-type RA cells (Figure 3a).

To further explore the function of RAYM_01812 in complement escape, duck serum – with or without preheating at 56°C for 30 min – was incubated with different concentrations of RAYM_01812 protein for 30 min at 37°C in HBSS. Subsequently, DH5α cells were added and incubated for 30 min at 37°C. Then, the cells were 10-fold serially diluted, spread onto LB agar plates, and incubated at 37°C for 24 h. The results showed that the survival rate of *E. coli* DH5α decreased significantly to 5.1% after interaction with 2% duck serum and increased significantly to 13.6%, 56.9%, and 97.2% respectively after sequential incubation with 10 µg, 50 µg, and 100 µg of protein. These findings suggest that the serine protease RAYM_01812 may inhibit the activation of the complement system, enabling *E. coli* to evade the immune response (Figure 3b).

To verify whether RAYM_01812 inhibits complement activation through IgY cleavage, IgY was incubated with 1, 2, and 10 μM RAYM_01812 at 37°C. Compared to RAYM_01812 incubated with PMSF, which served as a control, SDS-PAGE indicated that RAYM_01812 cleaved IgY in a concentration-dependent manner (Figure 3c).

Considering the importance of MAC formation – the terminal phase of complement activation – in Gram-negative bacteria killing, we then analyzed MAC deposition on the surface of RA-YM and ΔRAYM by SDS-PAGE. Following incubation with 5% final concentration of healthy duck serum at 37°C, the bacteria activated the complement reaction in duck serum, resulting in a gradual decrease of C9 monomers and a gradual increase of MAC formation. Interestingly, whereas the deposition of multimers appeared at 40 min in RA-YM, it did so at 20 min in ΔRAYM. These results underscore the inhibitory effect of RAYM_01812 on surface MAC formation (Figure 3d).

### RAYM_01812 cleaves C3a/C5a to inhibit monocyte chemotaxis

**Figure 4.**
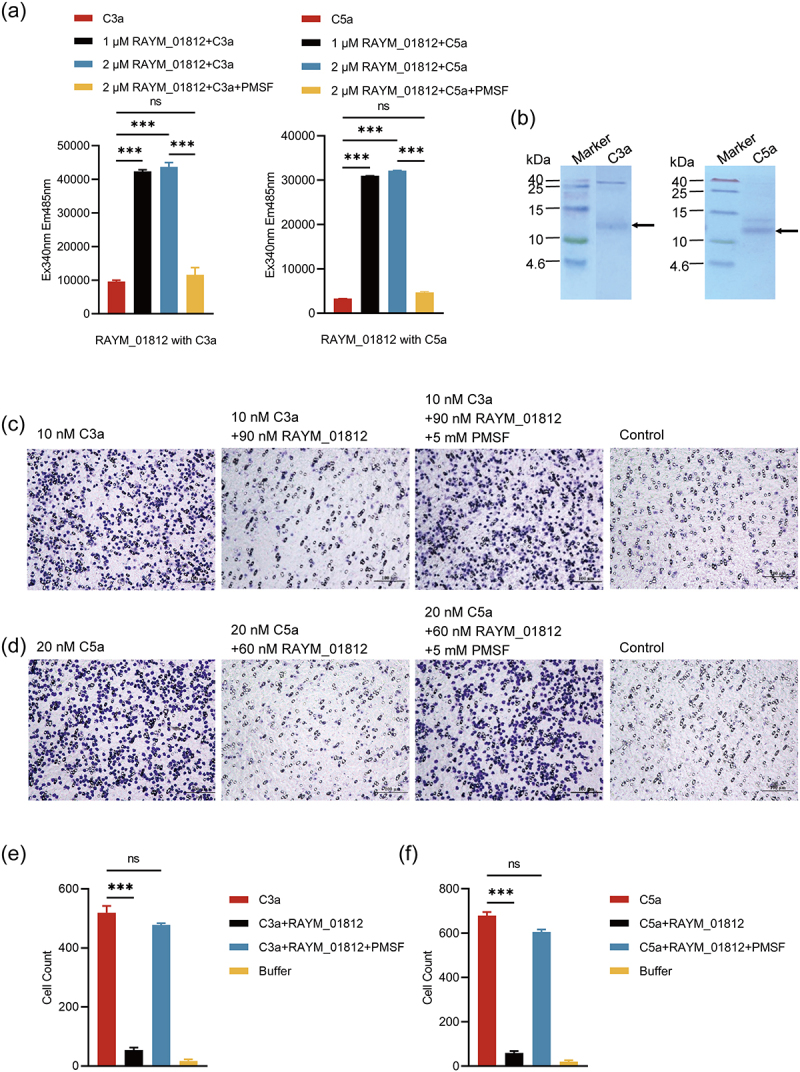
Effect of RAYM_01812 on C3a/C5a chemotaxis. (a) FRET validation of the cleavage action of RAYM_01812 on C3a and C5a. The values measured without RAYM_01812 were used as negative controls and the groups with 5 mm PMSF added were used as experimental controls. (b) SDS-page detection of purified C3a and C5a proteins. Lanes: M, molecular weight marker; lanes 1 were C3a, lanes 2 were C5a. Note: observed C3a or C5a are indicated by arrow (→). (c) Effect of RAYM_01812 on the chemotaxis of C3a. (d) Effect of RAYM_01812 on the chemotaxis of C5a. (e) Significance analysis of the effect of RAYM_01812 on the chemotaxis of C3a. (f) Significance analysis of the effect of RAYM_01812 on the chemotaxis of C5a. Data were analyzed with Student’ s t-test (*p < 0.05; **p < 0.01; ***p < 0.001; ns, not significant).

Considering that complement C3a and C5a chemokines play important roles in the inflammatory response through phagocytosis regulation, many pathogenic bacteria express C5a-targeting proteases to escape host complement immunity [27]. Thus, we verified the capacity of RAYM_01812 to cleave C3a and C5a by fluorescence resonance energy transfer (FRET) and monocyte chemotaxis assays. First, we synthesized short duck fluorescently labelled C3a and C5a peptides as the substrates for the cleavage assay and evidenced with FRET that RAYM_01812 cleaves C3a/C5a in a concentration-dependent manner (Figure 4a).

Next, to verify whether RAYM_01812-mediated cleavage of C3a and C5a would affect chemotaxis, we performed *in vitro* monocyte chemotaxis experiments with purified C3a and C5a proteins (Figure 4b) and evidenced a significantly reduced chemotaxis ability in the presence of RAYM_01812 (Figure 4c–f). These observations evidenced the ability of RAYM_01812 to inhibit C3a- and C5a-mediated chemotaxis.

### RAYM_01812 deletion attenuates RA virulence

**Figure 5.**
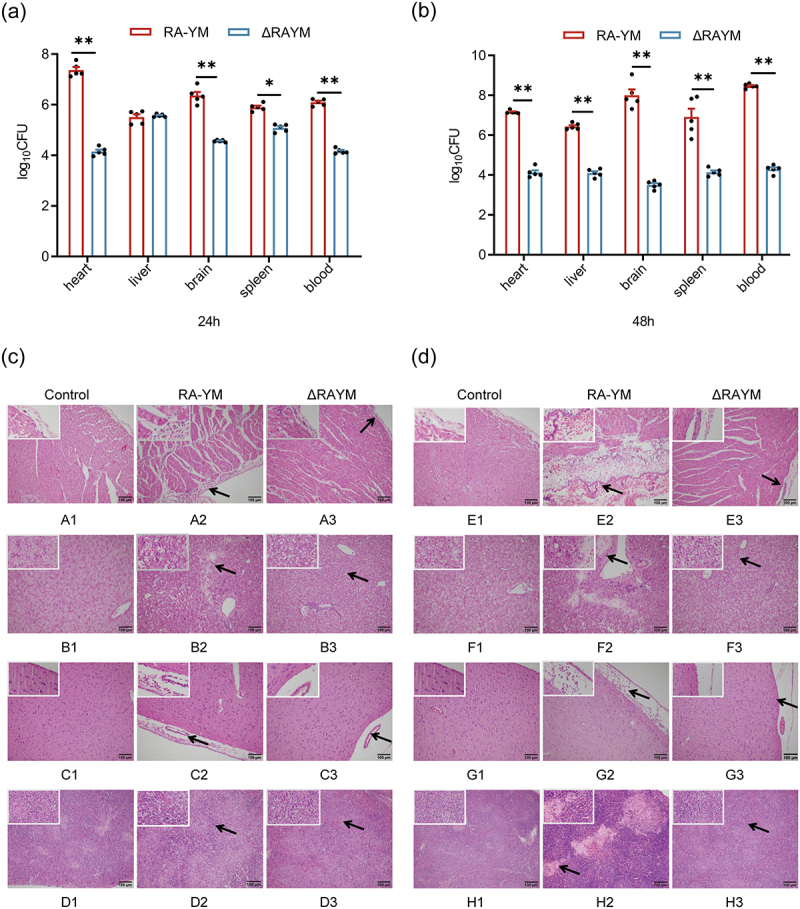
Determination of blood and tissue load of ducklings infected with *R. anatipestifer* RA-YM or *R. anatipestifer* ΔRAYM. Tissue burden in groups infected with RA-YM or ΔRAYM measured at 24 h (a) and 48 h (b) post challenge. Data represent mean ± standard deviation of five animals. Data were analyzed with the Student’s t-test (*p < 0.05, ** *p* < 0.01). Tissue specimens were analyzed at 24 h (c) and 48 h (d) post challenge.

The LD_50_ of the mutant ΔRAYM strain was 1.09 × 10^3^-fold lower compared to the parent RA-YM and CΔRAYM complemented strain (5.83 × 10^7^ CFU versus 5.33 × 10^4^ CFU or 9.77 × 10^6^ CFU, respectively) (Table 2). These findings indicate that the virulence of ΔRAYM was potently suppressed and could be rescued by complementation. To examine the invasiveness of the bacteria, the bacterial load in the blood, spleen, liver, heart, and brain was quantified. Compared to RA-YM, the bacterial burden of ΔRAYM in the blood, spleen, and liver was much lower at 24 and 48 h post-challenge (Figure 5a,b). Also, RA-YM-infected animals showed obvious congestion in the hepatic sinusoid and central vein as well as fatty degeneration of hepatocytes. Moreover, the hepatic sinusoid and the subarachnoid space showed a large quantity of epicardial fibrin exudate and inflammatory cells together with splenic white pulp lymphocyte proliferation. Of note, this phenotype was greatly attenuated in the ΔRAYM-infected groups, both 24 and 48 h post-challenge (Figure 5c,d).

**Table 2.** Death of ducklings inoculated the ΔRAYM strain, CΔRAYM strain and RA-YM strain.

Dose (CFU/0.5 mL)	Survived/Total		
	RA-YM	ΔRAYM	CΔRAYM
1.14 × 10^9^	12/12	10/12	12/12
1.14 × 10^8^	12/12	5/12	12/12
1.14 × 10^7^	12/12	4/12	12/12
1.14 × 10^6^	12/12	3/12	10/12
1.14 × 10^5^	8/12	0/12	7/12

Compared to control, the RA-YM wild strain-infected group had thickened epicardium, the adjacent myocardium was degenerated and necrotic, and the interstitium was widened and filled with a large number of heterophilic granulocytes, macrophages, lymphocytes, and plasma cells after 24 h of infection (Figure 5c A2). At 48 h after infection, the epicardium of the RA-YM wild strain-infected group adhered to a large amount of fibrous-like exudate and a large amount of inflammatory cells and necrotic cell debris, while the interstitium was characterized by severe hemorrhage in the tissue (Figure 5d E2). The liver structure of the RA-YM wild strain-infected group was disrupted and had a large number of degenerated and necrotic hepatocytes, while foci of marked liquefied necrosis formed around the confluent area (Figure 5c B2). At 48 h after infection, hepatocellular steatosis was severe and large areas of necrosis formed within the tissue with large numbers of necrotic hepatocytes (Figure 5d F2). The submeningeal space of the RA-YM wild strain-infected group was widened and contained a large amount of eosinophilic red fluid as well as heterophilic granulocytes, lymphocytes, and macrophages at 24 h after infection. The brain parenchyma was characterized by neuronal cell degeneration and necrosis with foci of liquefied necrosis and small hemorrhagic foci (Figure 5c C2). At 48 h after infection, the submeningeal space contained a large number of heterophilic granulocytes, macrophages, lymphocytes, and plasma cells. The perivascular space was widened, suggesting the presence of edema (Figure 5d G2). In the spleen of the RA-YM wild strain-infected group, lymphocytes were reduced and histiocytes were hyperplastic at 24 h after infection (Figure 5c D2). At 48 h after infection, the splenic tissue was characterized by a large number of degenerated and necrotic lymphocytes with large foci of necrotic areas (Figure 5d H2). Although the symptoms of the infected group of the ΔRAYM gene deletion strain were similar to those of the wild strain, the symptoms were milder (Figure 5c A3,B3,C3,D3). Compared to the control group, the ΔRAYM gene deletion strain infected group had milder pathological symptoms (Figure 5c E3,F3,G3,H3). Compared to the control group, the RA-YM group showed more severe pathological changes at both time points after infection with the most severe lesions at 48 h post-infection. In contrast, the ΔRAYM gene deletion strain group showed only mild symptoms after infection. Of note, the pathologic changes were more pronounced at 24 h compared to 48 h post-infection.

## Discussion

Bacterial proteases are important virulence factors mediating bacterial pathogenicity [28,29]. Serine proteases – including more than one-third of all proteases described such as trypsin, chymotrypsin, elastase, and subtilisin – play important physiological roles in biological organisms. Of these, subtilisin – representing the second largest family of serine proteases – contain a catalytic triad consisting of histidine (His), aspartic acid (Asp), and serine (Ser) in the active center [30]. Previously, substitution of any of the catalytic triad residues (Asp126, His158, or Ser410) of catalytically active SspA of *R. anatipestifer* by alanine (Ala) was shown to abolish its protease activity, confirming the conservatism of the catalytic triplet in serine protease evolution [22]. In this study, we first mutated the active site Ser410 of RAYM_01812 to Ala and demonstrated that an RA strain carrying the non-functional protease was unable to liquefy gelatin (Figure 1e). Also, we evidenced impaired enzyme activity in the culture filtrate of ΔRAYM compared to a wild-type and complementary RA-YM strain (Table 1), indicating that RAYM_01812 is the only extracellular gelatinase of RA.

Maturation of subtilisin proteases occurs through auto-proteolysis without assistance of exogenous proteases. Upon its expression, subtilisin contains an N-terminal propeptide (pro-subtilisin) which acts as an intramolecular chaperone assisting in proper folding while meanwhile preventing activation of the mature enzyme through inhibition of the catalytic domain. Of note, some subtilisin-like serine proteases contain both an N-terminal and C-terminal propeptide. Following its secretion into the extracellular space, cleavage of the propeptide by the pro-subtilisin self-cleavage form initially generates the post-cleavage complex – consisting of the propeptide and the mature enzyme – and subsequent release of the active protease.

Through construction of truncation mutants, we analyzed the functions of the linker, the β-folded sheet, and CTD domains in RAYM_01812 secretion and maturation (Figure 2b,c). Even after 48 h incubation, the NC and NCL fragments of RAYM_01812 did not gain enzymatic activity, indicating the inability of these fragments to release the propeptide-mediated inhibitory effect on the catalytic domain. Consequently, these observations point to the crucial role of the β-folded sheet in N-terminal propeptide excision and thus protease maturation, while the linker- and CTD domains are seemingly redundant in this respect. Moreover, compared with NC and NCL, the NCLB- and NCLBT fragments degraded gelatin within 1 h (Figure 2d), further indicating the role of the β-folded sheet in protease folding and stability. Noteworthy, a similar β-jelly roll domain of the subtilisin-like serine protease TK-SP is not required for folding but rather mediates its hyperstability in a Ca^2+^-bound state [31,32].

Although the complement system is an important part of the innate immune system [12,33], only a few studies focused on duck complement immunity. In fact, the duck complement system is activated by three independent mechanisms including antibody (Ab)-mediated activation of the classical pathway as well as Ab-mediated and Ab-independent activation of the alternative pathway [34]. To reach its full capacity, divalent metal ions are essential for complement system activation [35,36]. For example, Mg^2+^ is a natural cofactor involved in the formation of C3 convertase within the alternative pathway [37]. Of note, in the presence of Ca^2+^ and Mg^2+^, the serum survival rates of ΔRAYM deleted strain were significantly lower compared to RA-YM and CΔRAYM. These findings are consistent with previous reports showing that the mutant strain Yb2ΔsspA exhibited reduced serum resistance compared to the wild-type strain *R. anatipestifer* Yb2 [22]. However, upon EGTA-mediated Mg^2+^ chelation, the serum survival rates of ΔRAYM strain increased about ten-fold from 8.8% to 82.6% (Figure 3a). These results indicate the essential role of the alternative pathway in the serum bactericidal effect; an observation which was previously described in *Edwardsiella tarda*, *E. coli*, and *R. anatipestifer* [36,38–40].

Immunoglobulin-degrading enzymes – a set of proteins expressed by some pathogenic bacteria – cleave extracellular Ig as well as the C3 and C5 anaphylatoxins. For example, *Streptococcus pyogenes* interferes with opsonophagocytosis through secreted cysteine proteases IdeS (also known as Mac-1 or MspA)-, SpeB-, and endoglycosidase EndoS-mediated cleavage of bacterial surface-bound IgG. Also, *Porphyromonas gingivalis* gingipain-mediated cleavage of complement component C3 results in the formation and subsequent degradation of C3a and C3b fragments which ultimately prevents the formation of C5 convertase. In parallel, gingipains also cleave C5 into bioactive C5a and C5b fragments and prevent MAC formation through C5b degradation [41]. Based on these data, we reasoned that the RAYM_01812-mediated cleavage of IgY observed in the current study could affect IgY-mediated immune response by preventing MAC formation. Also, it was previously shown that the cleavage of only one heavy chain per immunoglobulin molecule in the hinge region is in fact sufficient to inactivate it [42,43].

In the current study, we evidenced that RAYM_01812 can cleave C5a and C3a, as verified by FRET experiments (Figure 4a). C3a- and C5a-mediated complement activation results in chemotaxis and chemical induction of marrow cells such as neutrophils [44], respectively. Thus, we evidenced that RAYM_01812 could significantly inhibit the chemotaxis of C5a and C3a through monocyte chemotaxis tests (Figure 4c,d). Of note, similar mechanisms allowing escape of complement immunity through inhibition of C5a and C3a chemotaxis were previously described in other pathogenic microorganisms [45]. For example, *Pseudomonas* escapes complement immunity through cleavage of C3a with elastase (PaE) and alkaline protease (PaAP) [46]. Also, *Streptococcus pneumoniae* and *Streptococcus agalactis* prevent C5a chemotaxis through C5a peptidase-induced cleavage of complement C5a [47–50]. Based on these observations, we speculate that RAYM_01812-mediated immune escape operates by cleavage of the complement activation products C5a and C3a and subsequent inhibition of C5a and C3a chemotaxis.

In the present study, we showed that the lethality of the ΔRAYM mutant strain was more than 1000 times lower, while the bacterial numbers of the mutant in heart, liver, spleen, brain, and blood were on average 3 orders of magnitude less than those infected with the wild-type parent strain compared to the wild-type strain RA-YM. Pathological tissue sections showed that the symptoms of the infected group of the ΔRAYM gene deletion strain were similar to those of the wild-type strain, but were less severe (Table 2, Figure 5). This corroborates previous results showing that the bacterial load in the blood of ducks infected with the Yb2ΔsspA strain was significantly lower than those infected with the wild-type strain Yb2, while the Yb2ΔsspA strain exhibited increased adhesion and invasion capacity, alongside attenuated virulence [22]. These observations further suggest that RAYM_01812 mediates *R. anatipestifer* pathogenicity. It is worth noting that pathology slides have observational limitations, and sampling is susceptible to subjective factors, so accuracy in sampling is crucial.

In summary, our results demonstrate that RAYM_01812 is an important virulence factor of *R. anatipestifer*. In fact, whereas RAYM_01812 mediates resistance of the pathogen to the host serum defense mechanism, the ΔRAYM mutant lacks gelatinase activity and results in attenuated virulence. Moreover, RAYM_01812 enables evasion of the complement system through protease-mediated degradation of complement components and subsequent MAC assembly inhibition. A better insight in the intimate interplay between complement system-mediated microbial clearance as well as the bacterial escape mechanisms is crucial to understand microbial virulence. Ultimately, this will lead us to establish novel antibiotic- and vaccine targets in bacterial pathogens thus limiting the chances of bacterial resistance towards commonly used antibiotics.

## Supplementary Material

Supplemental_Material _ Tables _242702962.docx

## Data Availability

The raw data that support the findings of this study have been uploaded to Figshare repository (https://figshare.com/s/475f3802a7bbe0bcff1a). The gene sequences have been deposited to NCBI (https://www.ncbi.nlm.nih.gov/protein/315023007) with the GenBank accession number EFT36020.1 Further inquiries can be directed to the corresponding author.
