# Supplementary material for: Serine protease RAYM_01812 (SspA) inhibits complement-mediated killing and monocyte chemotaxis and contributes to virulence of Riemerella anatipestifer in ducks
Source: Virulence. 2024 Oct 25;15(1):2421219. doi: 10.1080/21505594.2024.2421219 (PMC11540087; doi:10.1080/21505594.2024.2421219)
Supplement: Supplemental_Material _ Tables _242702962.docx [file KVIR_A_2421219_SM1979.docx]

**Supplementary Table 1 Strains, plasmids, and primers used in this study.**

| **Strains, plasmids, or primers** | **Descriptions** | **Source of reference** |
| --- | --- | --- |
| **Strains** |  |  |
| RA-YM | *Riemerella anatipestifer* wild-type strain, serotype 1 | [1] |
| ΔRAYM | RAYM_01812 gene deletion mutant strain, Spc^R^ | This study |
| CΔRAYM | Complemented CΔRAYM strain, Spc^R^, Amp^R^ | This study |
| *E.coli* DH5α | Competent cell | [2] |
| *E.coli* ꭓ7213 | Competent cell | [3] |
| *E.coli* BL21(DE3) | Expression cell | [4] |
| **Plasmids** |  |  |
| pMD18-T | TA cloning vector | Takara |
| pRE112 | Suicide vector | [5] |
| pRE112-LSR | Suicide vector | This study |
| RA-JX | Wild type plasmid of RA | [6] |
| pRES-JX-bla | Shuttle vector | [7] |
| pRES-C01812 | Shuttle vector | This study |
| pET-28a | Expression vector | Novagen |
| pET-28a-01812(NCLβT) | Expression vector | This study |
| pET-28a-NC | Expression vector | This study |
| pET-28a-NCL | Expression vector | This study |
| pET-28a-NCLβ | Expression vector | This study |
| pET-28a-NCT | Expression vector | This study |
| pET-28a-NCLT | Expression vector | This study |
| pET-28a-CD | Expression vector | This study |
| pET-28a-β | Expression vector | This study |
| pET-28a-C3a | Expression vector | This study |
| pET-28a-C5a | Expression vector | This study |
| pET-28a-01812(S410A ) | Expression vector | This study |
| **Primers (5’-3’)** |  |  |
| *spc-*F1 | CAGTGGAACGAAAACTCACGTTAAG | This study |
| *spc-*F2 | CAGTAGTTTTAAAAGTAAGCACCTG | This study |
| *01812*-F1 | GGGGTACCTAGATTTATAGCCTAATGGGT | This study |
| *01812*-F2 | ACGTGAGTTTTCGTTCCACTGTTGAGCAGCAATAACTCCACT | This study |
| *01812*-R1 | TGCTTACTTTTAAAACTACTGCAGGTTTGACACCAGGTGTG | This study |
| *01812*-R2 | CGAGCTCATATTACTTTTGGAACGCCT | This study |
| *01812-*C1 | ACATGCATGCTTACCCGTAGATTTATAG | This study |
| *01812-*C2 | CGTCTAGATTACTTCTTGATAAATTTCT | This study |
| *01812-*N1 | AACACGATGACTTTGGAGG | This study |
| *01812-*N2  NC-F  NC-R  NCL-F  NCL-R  NCLβ-F  NCLβ-R  NCLβT-F  NCLβT-R  NCT-F1  NCT-R1  NCT-F2  NCT-R2  NCLT-F1  NCLT-R1  NCLT-F2  NCLT-R2  CD-F  CD-R  β-F  β-R  C3a-F  C3a-R  C5a-F  C5a-R  S410A-F  S410A-R | CACTTCCATAAGCCCACTC  CGCGGATCCCAAAACCAAAACACATCTCTTGAG  CCGCTCGAGCGAATGTACCAAAAGTGTTTTCGCCGATGCTGCATCTAA  CGCGGATCCCAAAACCAAAACACATCTCTTGAG  CCGCTCGAGCTTTACAAGAAGTTCTGCTCCTTTTTGTG  CGCGGATCCCAAAACCAAAACACATCTCTTGAG  CCGCTCGAGTAATACAATCTCCGTATATCCAGAAACCATAATGCT  CGCGGATCCCAAAACCAAAACACATCTCTTGAG  CCGCTCGAGCTTCTTGATAAATTTCTTAGTAACCTCACCG  CGCGGATCCCAAAACCAAAACACATCTCTTGAG  CCGCTCGAGCTTCTTGATAAATTTCTTAGTAACCTCACCG  CGAAAACACTTTTGGTACATACGACTCAGTCTATTGATAAGCCATC  TTATCAATAGACTGAGTCGTATGTACCAAAAGTGTTTTCGCCGATG  CGCGGATCCCAAAACCAAAACACATCTCTTGAG  CCGCTCGAGCTTCTTGATAAATTTCTTAGTAACCTCACCG  GAGCAGAACTTCTTGTAAAGACGACTCAGTCTATTGATAAGCCATCG  TTATCAATAGACTGAGTCGTCTTTACAAGAAGTTCTGCTCCTTTTTGT  CGCGGATCCGATTTTATTCAAGATGGTAGAGTTTCAGGACTTGCAAAA  CCGCTCGAGCGAATGTACCAAAAGTGTTTTCGCCGATGCTGCATCTAA  CGCGGATCCAAAAGTAACGACGAAATTATTTTTGAAACTAAGC  CCGCTCGAGTAATACAATCTCCGTATATCCAGAAACCATAATGCT  CGCGGATCCAGCCTGCAGCTCATCGAGTAC  CCCAAGCTTGTCGAGCCAGCTCGAGATAGG  CGCGGATCCGACTTCCAGGAGCGAATACA  CCCAAGCTTGTCTTGCCAATATTAGCAGCT  TATGGAAGTGGAACTGCTTATTCAGCTCCTC  GAGGAGCTGAATAAGCAGTTCCACTTCCA | This study  This study  This study  This study  This study  This study  This study  This study  This study  This study  This study  This study  This study  This study  This study  This study  This study  This study  This study  This study  This study  This study  This study  This study  This study  This study  This study |

^R^Resistance.

**Supplementary Table 2 Primers for fluorogenic substrate**

| Substrate name | Sequence  （N’-C’） | Modification | solvent | Molecular Weight | Purity |
| --- | --- | --- | --- | --- | --- |
| DUCK-C3a | GIREQKQRESYLELAR | DABCYL/Glu(EDANS) | DMSO | 2604.91 | ≥95.5％ |
| DUCK-C5a | NRLREEEPNKLLILAR | DABCYL/Glu(EDANS) | DMSO | 2592.98 | ≥95.6％ |

**Supplementary Table 3 Comparison of the biochemical characteristics between ΔRAYM strain, CΔRAYM strain and RA-YM strain**

| strain | Glu | Suc | Man | Ara | Lac | Mal | Xyl | Car | Cit | Sor | H_2_S | Gel |
| --- | --- | --- | --- | --- | --- | --- | --- | --- | --- | --- | --- | --- |
| RA-YM | — | — | — | — | — | — | — | — | — | — | — | + |
| ΔRAYM | — | — | — | — | — | — | — | — | — | — | — | — |
| CΔRAYM | — | — | — | — | — | — | — | — | — | — | — | + |

Note: Glu, glucose; Suc, sucrose; Man, mannitol; Ara, arabinose; Lac, lactose; Mal, maltose; Xyl, Xylose; Car, carbamide; Cit, citrate; Sor, sorbitol; Gel, gelatin; -, Negative; +, Positive

**References**

1. Zhou Z, Zheng J, Tian W, Li J, Zhang W, Zhang J, Meng X, Hu S, Bi D, Li Z. Identification of *Riemerella anatipestifer* genes differentially expressed in infected duck livers by the selective capture of transcribed sequences technique. Avian Pathol. 2009 Aug;38(4):321-9. doi: 10.1080/03079450903071311. PMID: 19937518.
2. Hanahan D, Jessee J, Bloom FR. Plasmid transformation of *Escherichia coli* and other bacteria. Methods Enzymol. 1991;204:63-113. doi: 10.1016/0076-6879(91)04006-a. PMID: 1943786.
3. Roland K, Curtiss R 3rd, Sizemore D. Construction and evaluation of a delta cya delta crp *Salmonella typhimurium* strain expressing avian pathogenic *Escherichia coli* O78 LPS as a vaccine to prevent airsacculitis in chickens. Avian Dis. 1999 Jul-Sep;43(3):429-441. PMID: 10494411.
4. Miroux B, Walker JE. Over-production of proteins in *Escherichia coli*: mutant hosts that allow synthesis of some membrane proteins and globular proteins at high levels. J Mol Biol. 1996 Jul 19;260(3):289-298. doi: 10.1006/jmbi.1996.0399. PMID: 8757792.
5. Edwards RA, Keller LH, Schifferli DM. Improved allelic exchange vectors and their use to analyze 987P fimbria gene expression. Gene. 1998 Jan 30;207(2):149-57. doi: 10.1016/s0378-1119(97)00619-7. PMID: 9511756.
6. Guo Y, Hu D, Guo J, Li X, Guo J, Wang X, Xiao Y, Jin H, Liu M, Li Z, Bi D, Zhou Z. The Role of the Regulator Fur in Gene Regulation and Virulence of *Riemerella anatipestifer* Assessed Using an Unmarked Gene Deletion System. Front Cell Infect Microbiol. 2017 Aug 25;7:382. doi: 10.3389/fcimb.2017.00382. PMID: 28971067; PMCID: PMC5609570.
7. Guo Y, Hu D, Guo J, Wang T, Xiao Y, Wang X, Li S, Liu M, Li Z, Bi D, Zhou Z. *Riemerella anatipestifer* Type IX Secretion System Is Required for Virulence and Gelatinase Secretion. Front Microbiol. 2017 Dec 19;8:2553. doi: 10.3389/fmicb.2017.02553. PMID: 29312236; PMCID: PMC5742166.
